# A comparison of the structures of some 2- and 3-substituted chromone derivatives: a structural study on the importance of the secondary carboxamide backbone for the inhibitory activity of MAO-B

**DOI:** 10.1107/S2056989015017958

**Published:** 2015-10-03

**Authors:** Ligia R. Gomes, John Nicolson Low, Fernando Cagide, Alexandra Gaspar, Fernanda Borges

**Affiliations:** a FP-ENAS-Faculdade de Ciências de Saúde, Escola Superior de Saúde da UFP, Universidade Fernando Pessoa, Rua Carlos da Maia, 296, P-4200-150 Porto, Portugal; b REQUIMTE/Departamento de Química e Bioquímica, Faculdade de Ciências, Universidade do Porto, 4169-007 Porto, Portugal; c Department of Chemistry, University of Aberdeen, Meston Walk, Old Aberdeen, AB24 3UE, Scotland; d CIQUP/Departamento de Química e Bioquímica, Faculdade de Ciências, Universidade do Porto, 4169-007 Porto, Portugal

**Keywords:** crystal structure, chromones, pharmalogical activity, supra­molecular structure, hydrogen bonding

## Abstract

The crystal structures of the 3-substituted tertiary chromone carboxamide derivative, C_17_H_13_NO_3_, *N*-methyl-4-oxo-*N*-phenyl-4*H*-chromene-3-carboxamide (**1**), and the chromone carbonyl pyrrolidine derivatives, C_14_H_13_NO_3_, 3-(pyrrolidine-1-carbon­yl)-4*H*-chromen-4-one (**3**) and 2-(pyrrolidine-1-carbon­yl)-4*H*-chromen-4-one (**4**) have been determined. Their structural features are discussed and compared with similar compounds namely with respect to their MAO-B inhibitory activities. The chromone carboxamide presents a –*syn* conformation with the aromatic rings twisted with respect to each other [the dihedral angle between the mean planes of the chromone system and the exocyclic phenyl ring is 58.48 (8)°]. The pyrrolidine derivatives also display a significant twist: the dihedral angles between the chromone system and the best plane formed by the pyrrolidine atoms are 48.9 (2) and 23.97 (12)° in (**3**) and (**4**), respectively. Compound (**3**) shows a short C—H⋯O intra­molecular contact forming an *S*(7) ring. The supra­molecular structures for each compound are defined by weak C—H⋯O hydrogen bonds, which link the mol­ecules into chains and sheets. The Cambridge Structural Database gave 45 hits for compounds with a pyrrolidinecarbonyl group. A simple statistical analysis of their geometric parameters is made in order to compare them with those of the mol­ecules determined in the present work.

## Abstract 2

The structures of 2- and 3-substituted tertiary carboxamides and analogous pyrrolidine structures are compared.

## Chemical context   

Chromone (1-benzo­pyran-4-one) is the building block of a large family of natural and synthetic compounds of the utmost importance in medicinal chemistry (Gaspar *et al.*, 2014 ▸). Within this group of heterocycles, chromone carboxamide derivatives have been found to display inter­esting biological activities, namely as adenosine receptor ligands (Gaspar *et al.*, 2012 ▸) and as MAO-B inhibitors (Gaspar *et al.*, 2012 ▸; Gomes *et al.*, 2015*b* ▸; Cagide *et al.*, 2015 ▸). From the library synthesized so far, chromones (**1**)–(**6**) were selected for the present study, see Scheme. Previous data acquired on the development of new MAO-B inhibitors allowed us to conclude that 2-substituted chromones carboxamides based on the *N*-phenyl-4-oxo-4*H*-2-chromone carboxamide (**6**) skeleton have no significant IMAO-B activity whereas 3-substituted carboxamides based on the *N*-phenyl-4-oxo-4*H*-3-chromone carboxamide (**5**) core have been shown to be potent and selective inhibitors (Cagide *et al.*, 2015 ▸). Structure–activity relationship (SAR) studies revealed the significance of phenyl­carboxamide as a key structure. Structural investigations made so far show that the derivatives of (**5**) have very similar conformations and indicate that the displayed IMAO-B activity is mostly dependent on electronic factors modulated by the nature and position of the substituent group attached to the exocyclic phenyl substituent (Gomes *et al.*, 2015*a* ▸,*b* ▸). Despite this, those studies do not allow inferences to be made about (i) the importance of the carboxamide group, including the amidic hydrogen atom or (ii) the configuration of the amide due to the C–N rotamer, in the mol­ecular docking. Thus new compounds were synthesised and structurally characterized *viz. N*-methyl-4-oxo-*N*-phenyl-4*H*-chromene-3-carboxamide (**1**) and its isomer *N*-methyl-4-oxo-*N*-phenyl-4*H*-chromene-2-carboxamide (**2**), both tertiary carboxamides, as opposed to the secondary carboxamides (**5**) and (**6**) and 3-(pyrrolidine-1-carbon­yl)-4*H*-chromen-4-one (**3**) and 2-(pyrrolidine-1-carbon­yl)-4*H*-chromen-4-one (**4**), which instead of the carboxamide have a carbonyl pyrrolidine linked to the chromone (see Scheme). Compounds (**2**), (**5**) and (**6**), *N*-methyl-4-oxo-*N*-phenyl-4*H*-chromene-2-carboxamide, *N*-phenyl-4-oxo-4*H*-3-chromone carboxamide and *N*-phenyl-4-oxo-4*H*-2-chromone carboxamide, have previously been characterized by X-ray diffraction (Gomes *et al.* 2013 ▸, Cagide *et al.*, 2015 ▸ and Reis *et al.*, 2014 ▸, respectively). They will be used in this study for comparative purposes.
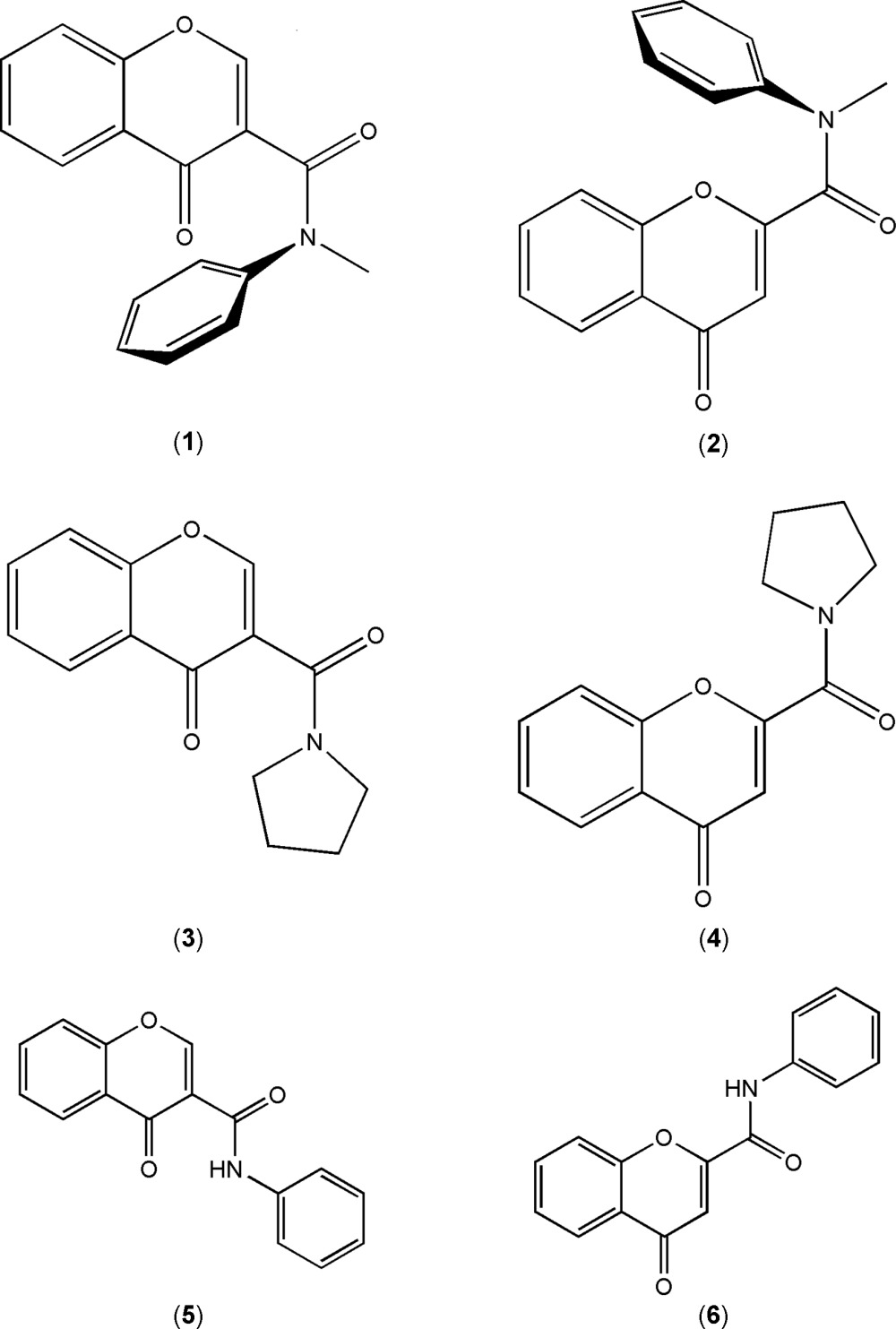


## Structural commentary   

### Mol­ecular Conformations   

**Figure 1:**
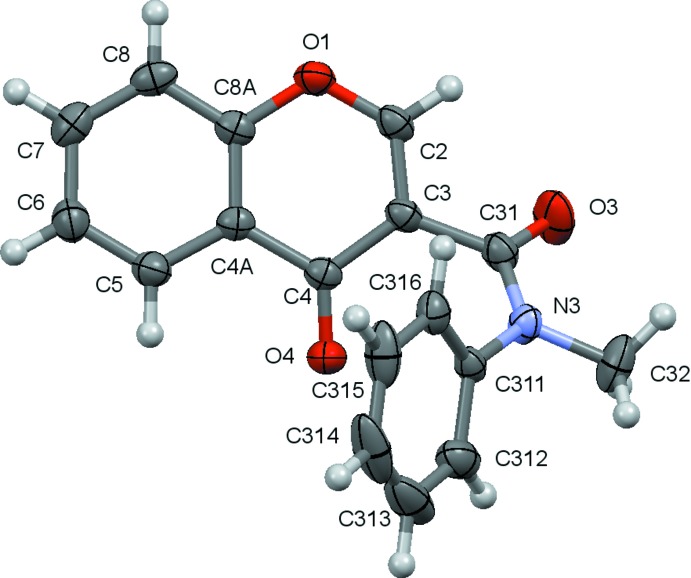
A view of the asymmetric unit of (**1**) with the atom-numbering scheme. Displacement ellipsoids are drawn at the 70% probability level.

As mentioned above, the compounds discussed in this work are presented in the Scheme. Compounds (**2**), (**5**) and (**6**) have previously been characterized. The ellipsoid plots for the remaining structures, *e.g*. for (**1**), (**3**) and (**4**), are given in Figs. 1 ▸–3 ▸ ▸. The results of the biological tests show that only (**5**) exhibits significant IMAO-B activity. Its isomer (**6**) is much less active while the remaining ones are inactive towards MAO-B, suggesting that substitution on position number 3 of the chromone is required and it must be a secondary carboxamide. As will be discussed, the presence of a tertiary amide induces significant conformational changes to the compounds that can explain the lack of activity for those compounds.

Compound (**1**) is a phenyl chromone carboxamide similar to (**5**) where the amidic hydrogen atom has been replaced by a methyl substituent. Since the nitro­gen atom of the amide tends to be planar due to the partial *sp* ^2^ hybridization of the C—N bond and, owing to the high rotational barrier around that bond, amides often exhibit –*anti*/–*syn* conformations with respect to the C–N rotamer. The inactive chromone carboxamides (**1**) and (**2**) present –*syn* conformations whereas chomone (**5**) (active) and (**6**) (inactive) are in the –*anti* form. In (**5**) and (**6**) the aromatic rings are roughly co-planar [dihedral angles between the mean planes of the aromatic rings are 10.77 (4) (Cagide *et al.*, 2015 ▸) and 6.57 (7)° (Reis *et al.*, 2013 ▸), respectively], while in compounds (**1**) and (**2**) the aromatic rings are twisted with respect to each other [dihedral angles between the mean planes of the chromone and the exocyclic phenyl rings are 58.48 (8) and 73.86 (5)° (Gomes *et al.*, 2013 ▸), respectively]. The twisting is probably driven by the minimization of steric hindrance that would arise from the prox­imity of the rings. 

Mol­ecules (**3**) and (**4**) present a chromone residue and an exocycle pyrrolidine ring separated by a carbonyl spacer. The pyrrolidine ring in (**3**) assumes a mostly envelope shape as it is puckered at C313, with θ(2) = 0.349 (5) Å and φ(2) = 78.8 (7)°. In (**4**) the pyrrolidine conformation is between an envelope and a half-chair, the ring being twisted at C213—C214, with θ(2) = 0.380 (3) Å and φ(2) = 91.0 (3)°. The inactive pyrrolidines (**3**) and (**4**) also display a high degree of torsion; the dihedral angles between the chromone and the best plane formed by the pyrrolidine atoms are 48.9 (2) and 23.97 (12)° respectively. A close analysis of the –(C=O)—N bond lengths for (**3**) [1.337 (4) Å] and (**4**) [1.340 (3) Å] shows that these values are comparable with those presented for the corresponding bonds in the carboxamides (**1**) [1.361 (2) Å] and (**2**) [1.3528 (14) Å], indicating partial *sp* ^2^ hybridization of the nitro­gen atom in (**3**) and (**4**). Furthermore, a search made in the Cambridge Structural Database (Groom & Allen, 2014 ▸) for structures containing the pyrrolidinecarbonyl unit (see *Database survey* section below) shows that the C—N distances range between 1.294 and 1.361 Å [the mean value is 1.335 (2) Å], suggesting that in carbonyl pyrrolidines the C—N bond displays partial hybrid­ization (Laursen *et al.*, 2013 ▸).

### Intra­molecular C—H⋯O bonding   

There is no intra­molecular hydrogen bonding in compounds (**1**) and (**2**). This contrasts with what occurs in (**5**) and (**6**) where, due to the presence of the imidic nitro­gen atom, the mol­ecules display N—H⋯O intra­molecular *S*(6) rings and, due to the –*anti* configuration, they present weak C_*arom*_—H⋯O hydrogen bonds (the carbonyl group of the amide acting as acceptor for the *ortho*-carbon atom of the benzyl ring), resulting in second *S*(6) rings (Cagide *et al.*, 2015 ▸; Reis *et al.*, 2014 ▸). In (**3**) there is a short intra­molecular contact C312—H312⋯O4 in which the pyrollidine carbon atom acts as a donor to the carbonyl oxygen atom, O4, of the chromone, forming an *S*(7) ring. The search of the CSD (Groom & Allen, 2014 ▸) described below found five mol­ecules containing a pyrrolidine carbonyl moiety that exhibit similar intra­molecular hydrogen bonding. In conclusion, apart from precluding the formation of an intra­molecular N—H⋯*A* bond, substitution of the amidic hydrogen atom by a methyl group in the carboxamide or the insertion of a carb­oxy­pyrrolidine unit in the chromone causes a large change in the conformational geometry of the mol­ecules that prevents a link to the active site of the MAO-B enzyme.

## Supra­molecular features   

Details of the hydrogen bonding are given in Tables 1 ▸, 2 ▸ and 3 ▸.

**Figure 4:**
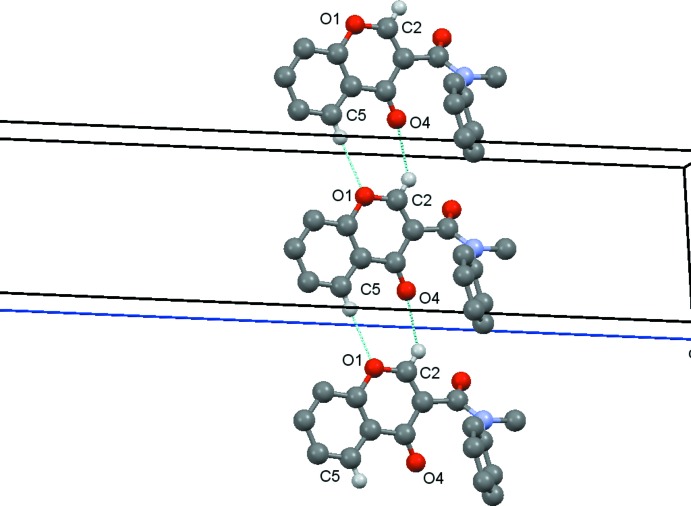
Compound (**1**): the chain of 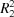
(8) rings running parallel to the *a* axis. Hydrogen atoms not involved in the hydrogen bonding are omitted.

**Figure 5:**
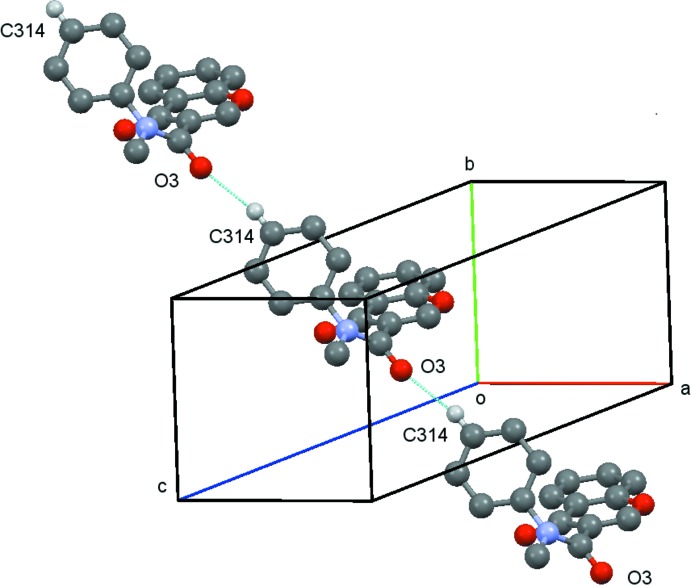
Compound (**1**): the chain formed by the C314—314⋯O3 hydrogen bond. Hydrogen atoms not involved in the hydrogen bonding are omitted.

**Figure 6:**
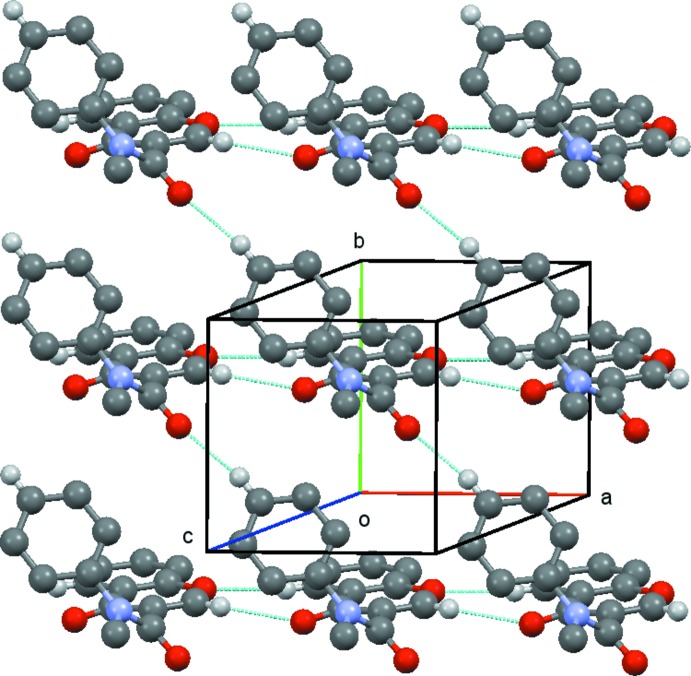
Compound (**1**): view of the sheets which lie parallel to [001] formed by the combination of the chains shown in Figs. 4 ▸ and 5 ▸. Hydrogen atoms not involved in the hydrogen bonding are omitted.

In compound (**1**) the C2—H2⋯O4(*x* + 1, y, z) and C5—H5⋯O1(*x* + 1, y, z) hydrogen bonds link the mol­ecules into 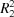
(8) rings which link the, mol­ecules into chains running parallel to the *a* axis, Fig. 4 ▸. These chains are then linked by the C314—314⋯O3(*x* − 1, *y* + 1, z) hydrogen bond, Fig. 5 ▸, to form sheets lying parallel to [001], Fig. 6 ▸. A centrosymmetric sheet inter­penetrates the first sheet, and these two sheets are linked by π–π stacking between the chromone rings [centroid–centroid distance = 3.557 (2) Å].

**Figure 7:**
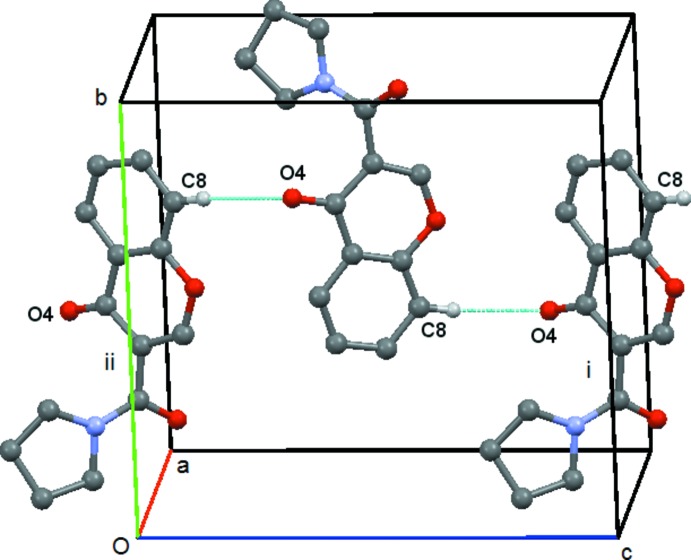
Compound (**3**): mol­ecular *C*(6) chain running parallel to the *c* axis. Mol­ecules i and ii are at (−*x* + 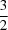, −*y* + 1, *z* + 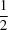) and (−*x* + 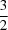, −*y* + 1, *z* − 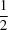), respectively. Hydrogen atoms not involved in the hydrogen bonding are omitted.

**Figure 9:**
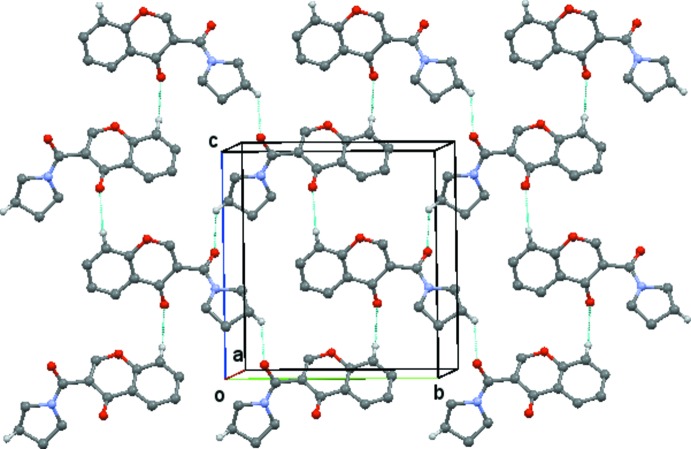
Compound (**3**): corrugated sheet in the *bc* plane formed by the inter­actions of the two *C*(6) chains shown in Figs. 7 ▸ and 8 ▸. Hydrogen atoms not involved in the hydrogen bonding are omitted.

**Figure 10:**
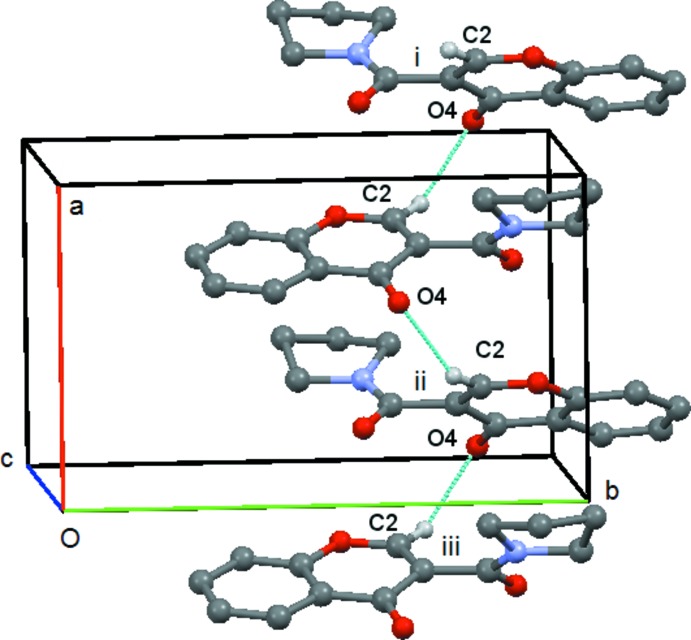
Compound (**3**): mol­ecular *C*(5) chain running parallel to the *a* axis. Mol­ecules i, ii and iii are at (*x* + 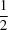, −*y* + 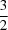, −*z* + 1), (*x* − 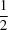, −*y* + 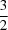, −*z* + 1) and (*x* − 1, *y*, *z*), respectively. Hydrogen atoms not involved in the hydrogen bonding are omitted.

**Figure 11:**
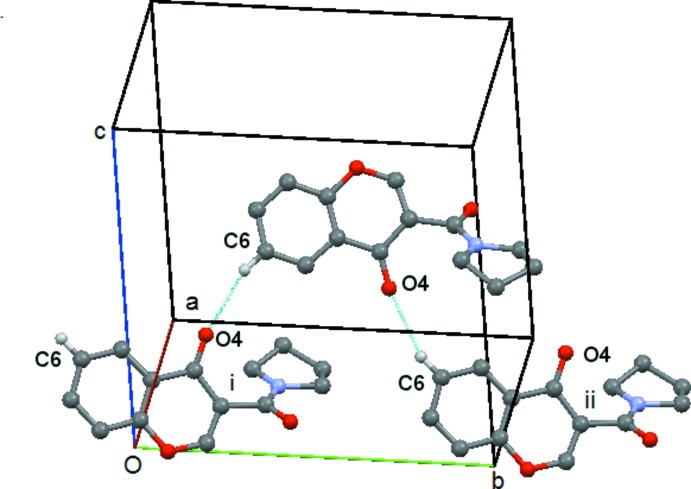
Compound (**3**): mol­ecular *C*(6) chain running parallel to the *b* axis. Mol­ecules i and ii are at (−*x* + 1, *y* − 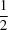, −*z* + 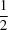) and (−*x* + 1, *y* + 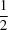, −*z* + 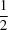), respectively. Hydrogen atoms not involved in the hydrogen bonding are omitted.

In compound (**3**) the mol­ecules are linked by C—H⋯O inter­actions and by C—H⋯π inter­actions. The C8—H8⋯O4(−*x* + 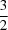, −*y* + 1, *z* + 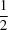) and C314—H31*E*⋯O3(−*x* + 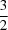, −*y* + 2, *z* − 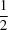) contacts both form *C*(6) chains running parallel to the *c axis* which are propagated by the twofold screw axes at (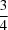, 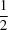, *z*) and (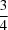, 1, *z*), respectively, Figs. 7 ▸ and 8 ▸. These combine to form a corrugated sheet in the *bc* plane, Fig. 9 ▸. The C2—H2⋯O4(*x* + 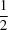, −*y* + 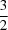, −*z* + 1) inter­action links the mol­ecules into *C*(5) chains running along the *a axis* propagated by the twofold screw axis at (*x*, 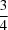, 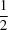), Fig. 10 ▸. The C6—H6⋯O4(−*x* + 1, *y* − 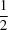, −*z* + 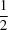) inter­action links the mol­ecules into *C*(6) chains running along the *b axis* which are propagated by the twofold screw axis at (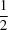, *y*, 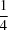), Fig. 11 ▸. There is also a C—H⋯π inter­action C313—H31*D*⋯*Cg*(*x* − 2, *y* + 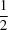, −*z* + 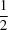). These inter­actions combine to form a complex three-dimensional network.

**Figure 12:**
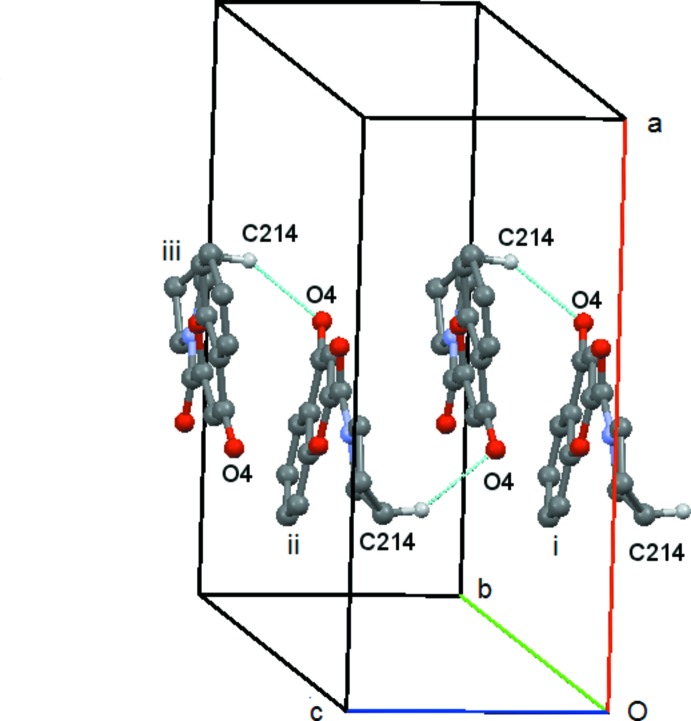
Compound (**4**): mol­ecular *C*(9) chain running parallel to the *c* axis. Mol­ecules i, ii and iii are at (−*x* + 1, −*y* + 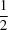, *z* − 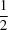), (−*x* + 1, −*y* + 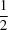, *z* + 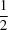) and (*x*, *y*, *z* + 1), respectively. Hydrogen atoms not involved in the hydrogen bonding are omitted.

**Figure 13:**
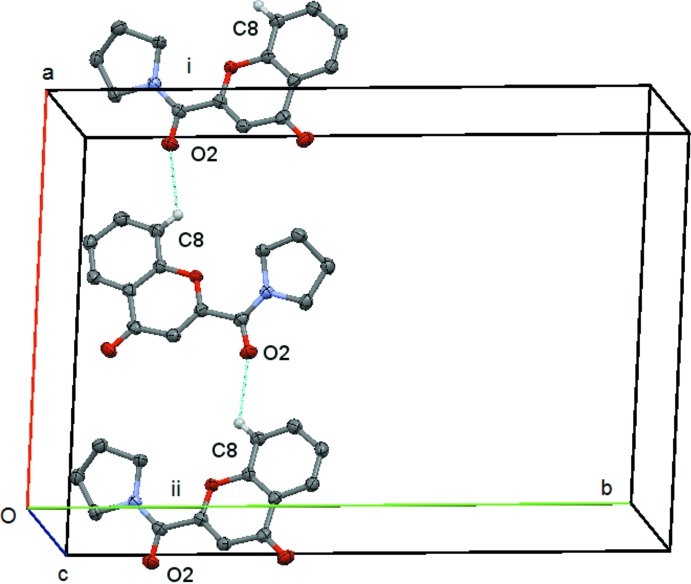
Compound (**4**): mol­ecular *C*(7) chain running parallel to the *a* axis. Mol­ecules i and ii are at (*x* + 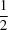, −*y* − 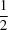, *z*) and (*x* − 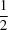, −*y* − 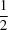, *z*), respectively. Hydrogen atoms not involved in the hydrogen bonding are omitted.

In compound (**4**) there is a short contact between C214—H21*C* and O4(−*x* + 1, −*y* + 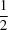, *z* − 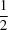). This forms a *C*(9) chain which runs along the *c axis*, propagated by the twofold screw axis at (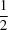, 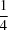, *z*), Fig. 12 ▸. There is also a short contact between C8—H8 and O2(*x* + 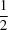, −*y* − 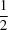, *z*) but in this case the angle at H8 is 121° and so this inter­action will be relatively weak. It forms a *C*(7) chain parallel to the *a axis* propagated by the glideplane at 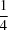 along the *b axis*, Fig. 13 ▸. There are no C—H⋯π or π–π inter­actions.

## Database survey   

A search of the Cambridge Structural Database (Groom & Allen, 2014 ▸) gave 45 hits for the pyrrolidinecarbonyl group for structures with *R* ≤ 0.10 (see supplementary data for the search fragment). The mean value for the C—O bond length was 1.235 (2) Å with a range of 1.209–1.282 Å. The values for (**3**) and (**4**) are 1.239 (4) and 1.230 (2) Å, respectively. The mean C—N bond length is 1.335 (2) Å with a range of 1.294–1.361Å. The values for (**3**) and (**4**) are 1.337 (4) and 1.340 (3) Å, respectively. The values for these compounds are close to the mean values in each case.

The torsion angles around the C(carbon­yl) and N(pyrrolidine) bond involving the carbonyl O atom lie in ranges between −9.15 and 8.023° with a mean value of close to zero and between −161.33 and 166.71° with a mean value close to 180° for both the C atoms attached to the N atom within the pyrolidine group. The respective torsion angles for (**3**) [−0.5 (5) and 171.9 (3)°] and those for (**4**) [1.1 (3) and −175.3 (2)°] are well within the ranges specified above.

**Figure 14:**
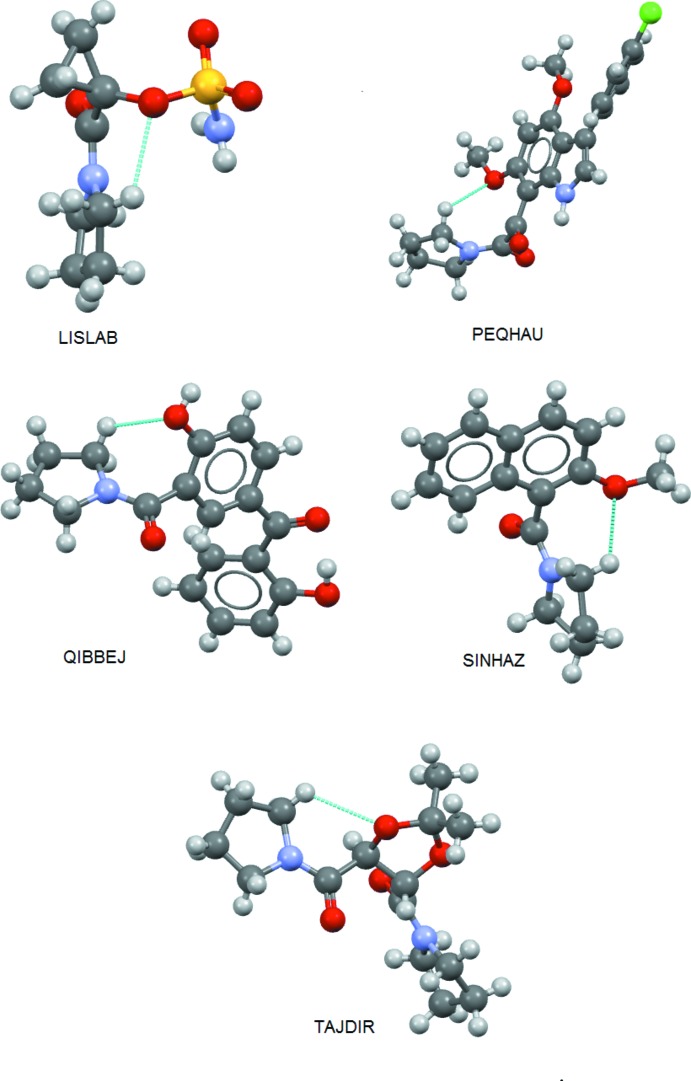
View of compounds in the CSD with C—H⋯O short intra­molecular contacts involving an *o-*pyrolidine hydrogen atom.

Intra­molecular C—H⋯O short contacts similar to that in (**3**) are found in five compounds in the CSD: LISLAB, 1-(1-pyrrolidinylcarbon­yl)cyclo­propyl sulfamate (Morin *et al.*, 2007 ▸), PEQHAU, 2-[3′-(4′′-chloro­phen­yl)-4′,6′-di­meth­oxy­indol-7′-yl]glyoxyl-1-pyrrolidine (Black *et al.*, 1997 ▸), QIBBEJ, [2-hy­droxy-5-(2-hy­droxy­benzo­yl)phen­yl](pyrrolidin-1-yl)methanone (Holtz *et al.*, 2007 ▸), SINHAZ, 2-meth­oxy-1-(1-pyrrolidinylcarbon­yl)naphthalene (Sakamoto *et al.*, 2007 ▸) and TAJDIR, (4*S*,5*S*)-4,5-bis­(pyrrolidinylcarbon­yl)-2,2-dimethyl-1,3-dioxolane (Garcia *et al.*, 1991 ▸), Fig. 10 ▸. In LISLAB and TAJDIR, *S*(6) rings are formed. In QIBBEJ and SINHAZ, an *S*(7) ring similar to that in (**2**) is formed. In PEQHAU, an *S*(8) ring is formed, Fig. 14 ▸.

## Synthesis and crystallization   

***N*** **-methyl-4-oxo-** ***N*** **-phenyl-4** ***H*** **-chromene-3-carboxamide**, (**1**) was synthesized in a low yield (10%) by a one-pot reaction using 4-oxo-4*H*-chromene-3-carb­oxy­lic acid as starting mat­erial. The activation of the carb­oxy­lic acid was obtained by the coupling reagent bromo­tripyrrolidino­phospho­nium hexa­fluoro­phosphate (PyBrOP) and the amide obtained by reacting the ester derivative with *N*-methyl­aniline. The crude product was purified by flash chromatography (ethyl acetate and ethyl acetate/ CH_2_Cl_2_ in an 4:1 ratio). Crystals suitable for X-ray diffraction were obtained from ethyl acetate.

**3-(Pyrrolidine-1-carbon­yl)-4** ***H*** **-chromen-4-one**, (**3**) and **2-(pyrrolidine-1-carbon­yl)-4** ***H*** **-chromen-4-one**, (**4**) were synthesized in moderate yields, 57% and 45%, by a one-pot reaction using 4-oxo-4*H*-chromene-3-carb­oxy­lic and 4-oxo-4*H-*chromene-2-carb­oxy­lic acids, respectively, as starting materials. The synthetic strategy encompasses the activation of the chromone carb­oxy­lic acids by reaction with phospho­rus(V) oxychloride with formation *in situ* of an acid chloride inter­mediate. The acid chlorides react with pyrrolidine giving the desired amides. Crystals suitable for X-ray diffraction for both compounds were obtained from a solution of CH_2_Cl_2_/*n*-hexane solvent 1:1, m.p. for (**3**): 421–426K; m.p. for (**4**): 382–386K.

## Refinement   

Crystal data, data collection and structure refinement details are summarized in Table 4 ▸. H atoms were treated as riding atoms with C—H(aromatic) = 0.95 Å and C—H(CH_2_) = 0.99 Å with *U*_iso_ = 1.2*U*_eq_(C), and C—H(meth­yl) = 0.98Å with *U*_iso_ = 1.5*U*_eq_(C). The methyl hydrogen atoms were generated in idealized positions and checked on a final difference map.

## Supplementary Material

Crystal structure: contains datablock(s) general, 1, 3, 4. DOI: 10.1107/S2056989015017958/lh5791sup1.cif

Structure factors: contains datablock(s) 1. DOI: 10.1107/S2056989015017958/lh57911sup2.hkl

Structure factors: contains datablock(s) 3. DOI: 10.1107/S2056989015017958/lh57913sup3.hkl

Structure factors: contains datablock(s) 4. DOI: 10.1107/S2056989015017958/lh57914sup4.hkl

Supporting information file. DOI: 10.1107/S2056989015017958/lh57911sup5.cml

Supporting information file. DOI: 10.1107/S2056989015017958/lh57913sup6.cml

Supporting information file. DOI: 10.1107/S2056989015017958/lh57914sup7.cml

CCDC references: 1427459, 1427458, 1025355

Additional supporting information:  crystallographic information; 3D view; checkCIF report

## Figures and Tables

**Figure 2:**
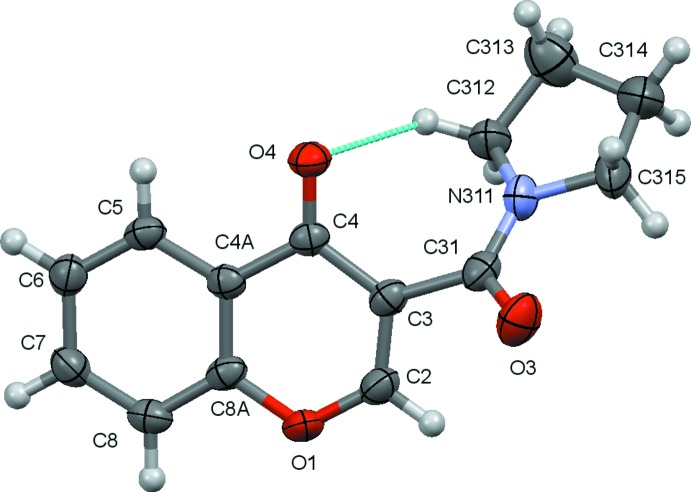
A view of the asymmetric unit of (**3**) with the atom-numbering scheme. Displacement ellipsoids are drawn at the 70% probability level.

**Figure 3:**
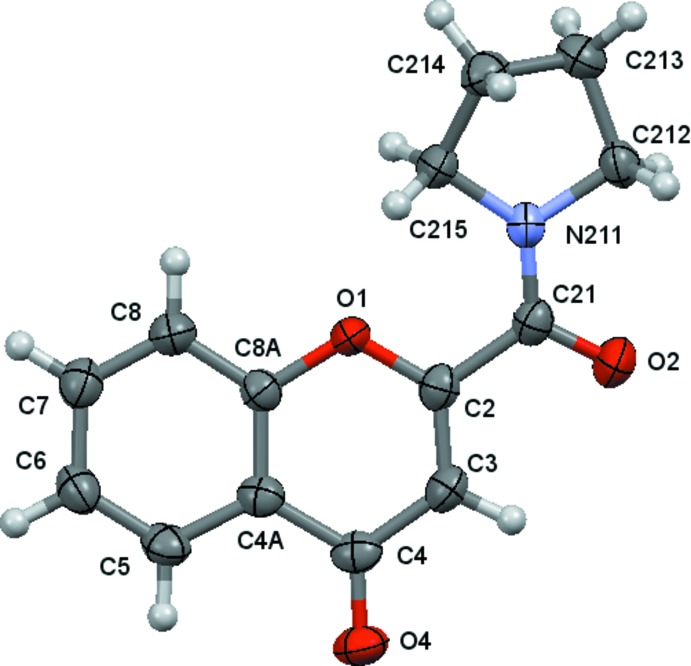
A view of the asymmetric unit of (**4**) with the atom-numbering scheme. Displacement ellipsoids are drawn at the 70% probability level.

**Figure 8:**
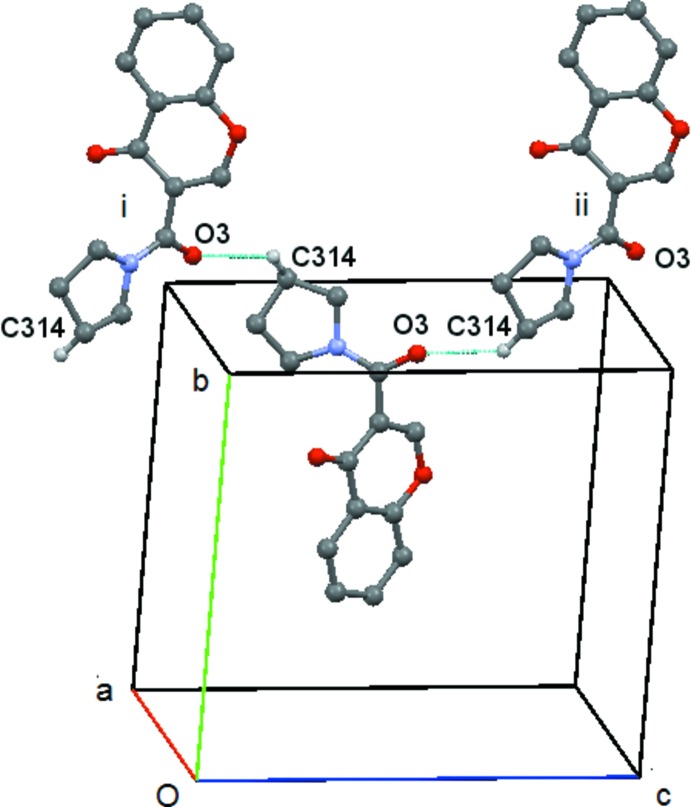
Compound (**3**): mol­ecular *C*(6) chain running parallel to the *c* axis. Mol­ecules i and ii are at (−*x* + 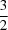, −*y* + 2, *z* − 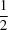) and (−*x* + 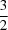, −*y* + 2, *z* + 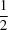), respectively. Hydrogen atoms not involved in the hydrogen bonding are omitted.

**Table 1:** Hydrogen-bond geometry (Å, °) for (**1**)

*D*—H⋯*A*	*D*—H	H⋯*A*	*D*⋯*A*	*D*—H⋯*A*
C2—H2⋯O4^i^	0.95	2.47	3.253 (3)	140
C5—H5⋯O1^ii^	0.95	2.49	3.432 (3)	172
C314—H314⋯O3^iii^	0.95	2.33	3.255 (3)	164

Symmetry codes: (i) 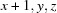; (ii) 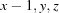; (iii) 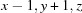.

**Table 2:** Hydrogen-bond geometry (Å, °) for (**3**) *Cg* is the centroid of the benzene ring C4*A*/C5–C8/C8*A*

*D*—H⋯*A*	*D*—H	H⋯*A*	*D*⋯*A*	*D*—H⋯*A*
C312—H31*A*⋯O4	0.99	2.29	3.082 (5)	136
C2—H2⋯O4^i^	0.95	2.47	3.338 (4)	152
C6—H6⋯O4^ii^	0.95	2.48	3.389 (4)	161
C8—H8⋯O4^iii^	0.95	2.57	3.514 (4)	170
C314—H31*E*⋯O3^iv^	0.99	2.42	3.128 (5)	128
C313—H31*D*⋯*Cg* ^v^	0.99	2.59	3.570 (6)	170

Symmetry codes: (i) 
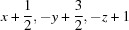
; (ii) 
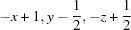
; (iii) 
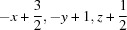
; (iv) 
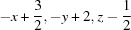
; (v) 
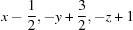
.

**Table 3:** Hydrogen-bond geometry (Å, °) for (**4**)

*D*—H⋯*A*	*D*—H	H⋯*A*	*D*⋯*A*	*D*—H⋯*A*
C8—H8⋯O2^i^	0.95	2.55	3.137 (3)	121
C214—H21*C*⋯O4^ii^	0.99	2.47	3.340 (3)	146

Symmetry codes: (i) 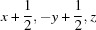; (ii) 
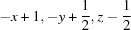
.

**Table 4:** Experimental details

	(**1**)	(**3**)	(**4**)
Crystal data			
Chemical formula	C_17_H_13_NO_3_	C_14_H_13_NO_3_	C_14_H_13_NO_3_
*M*_r_	279.28	243.25	243.25
Crystal system, space group	Monoclinic, *P*2_1_/*c*	Orthorhombic, *P*2_1_2_1_2_1_	Orthorhombic, *A* *b* *a*2
Temperature (K)	100	100	100
*a*, *b*, *c* (Å)	6.716 (4), 6.809 (4), 29.425 (17)	7.430 (3), 11.963 (6), 12.648 (6)	15.337 (6), 21.940 (8), 6.808 (3)
α, β, γ (°)	90, 94.784 (7), 90	90, 90, 90	90, 90, 90
*V* (Å^3^)	1340.9 (14)	1124.2 (9)	2290.8 (16)
*Z*	4	4	8
Radiation type	Mo *K*α	Mo *K*α	Mo *K*α
μ (mm^−1^)	0.10	0.10	0.10
Crystal size (mm)	0.25 × 0.07 × 0.06	0.22 × 0.07 × 0.03	0.50 × 0.04 × 0.02

Data collection			
Diffractometer	Rigaku Saturn724+	Rigaku Saturn724+	Rigaku Saturn724+
Absorption correction	Multi-scan (*CrystalClear-SM Expert*; Rigaku, 2012 ▸)	Multi-scan (*CrystalClear-SM Expert*; Rigaku, 2012 ▸)	Multi-scan (*CrystalClear-SM Expert*; Rigaku, 2012 ▸)
*T*_min_, *T*_max_	0.572, 1.000	0.978, 0.997	0.952, 0.998
No. of measured, independent and observed [*I* > 2σ(*I*)] reflections	6206, 3032, 2304	7547, 3294, 3066	9598, 3374, 3079
*R*_int_	0.043	0.031	0.034
(sin θ/λ)_max_ (Å^−1^)	0.648	0.731	0.731

Refinement			
*R*[*F* ^2^ > 2σ(*F* ^2^)], *wR*(*F* ^2^), *S*	0.056, 0.133, 1.07	0.069, 0.152, 1.07	0.048, 0.102, 1.11
No. of reflections	3032	3294	3374
No. of parameters	190	163	163
No. of restraints	0	0	1
H-atom treatment	H-atom parameters constrained	H-atom parameters constrained	H-atom parameters constrained
Δρ_max_, Δρ_min_ (e Å^−3^)	0.30, −0.25	0.51, −0.44	0.32, −0.21

Computer programs: *CrystalClear-SM Expert* (Rigaku, 2012 ▸), *SHELXS* (Sheldrick, 2015 ▸), *SHELXL2014/17* (Sheldrick, 2015 ▸) *PLATON* (Spek, 2009 ▸) *Flipper 25* (Oszlányi & Sütő, 2004 ▸) *OLEX2* (Dolomanov *et al.*, 2009 ▸, *OSCAIL* (McArdle *et al.*, 2004 ▸), *ShelXle* (Hübschle *et al.*, 2011 ▸) and *Mercury* (Macrae *et al.*, 2006 ▸).

## supplementary crystallographic information

### (1) N-Methyl-4-oxo-N-phenyl-4H-chromene-3-carboxamide Crystal data

**Table:**

C_17_H_13_NO_3_	*Z* = 4
*M**_r_* = 279.28	*F*(000) = 584
Monoclinic, *P*2_1_/*c*	*D*_x_ = 1.383 Mg m^−^^3^
*a* = 6.716 (4) Å	Mo *K*α radiation, λ = 0.71075 Å
*b* = 6.809 (4) Å	µ = 0.10 mm^−^^1^
*c* = 29.425 (17) Å	*T* = 100 K
β = 94.784 (7)°	Rod, colourless
*V* = 1340.9 (14) Å^3^	0.25 × 0.07 × 0.06 mm

### (1) N-Methyl-4-oxo-N-phenyl-4H-chromene-3-carboxamide Data collection

**Table:**

Rigaku Saturn724+ diffractometer	3032 independent reflections
Radiation source: Rotating Anode	2304 reflections with *I* > 2σ(*I*)
Confocal monochromator	*R*_int_ = 0.043
Detector resolution: 28.5714 pixels mm^-1^	θ_max_ = 27.4°, θ_min_ = 3.0°
profile data from ω–scans	*h* = −8→8
Absorption correction: multi-scan (*CrystalClear-SM Expert*; Rigaku, 2012)	*k* = −8→8
*T*_min_ = 0.572, *T*_max_ = 1.000	*l* = −17→38
6206 measured reflections	

### (1) N-Methyl-4-oxo-N-phenyl-4H-chromene-3-carboxamide Refinement

**Table:**

Refinement on *F*^2^	0 restraints
Least-squares matrix: full	Hydrogen site location: inferred from neighbouring sites
*R*[*F*^2^ > 2σ(*F*^2^)] = 0.056	H-atom parameters constrained
*wR*(*F*^2^) = 0.133	*w* = 1/[σ^2^(*F*_o_^2^) + (0.0336*P*)^2^ + 1.136*P*] where *P* = (*F*_o_^2^ + 2*F*_c_^2^)/3
*S* = 1.07	(Δ/σ)_max_ < 0.001
3032 reflections	Δρ_max_ = 0.30 e Å^−^^3^
190 parameters	Δρ_min_ = −0.25 e Å^−^^3^

### (1) N-Methyl-4-oxo-N-phenyl-4H-chromene-3-carboxamide Special details

**Table:**

Geometry. All e.s.d.'s (except the e.s.d. in the dihedral angle between two l.s. planes) are estimated using the full covariance matrix. The cell e.s.d.'s are taken into account individually in the estimation of e.s.d.'s in distances, angles and torsion angles; correlations between e.s.d.'s in cell parameters are only used when they are defined by crystal symmetry. An approximate (isotropic) treatment of cell e.s.d.'s is used for estimating e.s.d.'s involving l.s. planes.
Refinement. Refinement of *F*^2^ against ALL reflections. The weighted *R*-factor *wR* and goodness of fit *S* are based on *F*^2^, conventional *R*-factors *R* are based on *F*, with *F* set to zero for negative *F*^2^. The threshold expression of *F*^2^ > σ(*F*^2^) is used only for calculating *R*-factors(gt) *etc*. and is not relevant to the choice of reflections for refinement. *R*-factors based on *F*^2^ are statistically about twice as large as those based on *F*, and *R*- factors based on ALL data will be even larger.

### (1) N-Methyl-4-oxo-N-phenyl-4H-chromene-3-carboxamide Fractional atomic coordinates and isotropic or equivalent isotropic displacement parameters (Å^2^)

**Table:**

	*x*	*y*	*z*	*U*_iso_*/*U*_eq_	
O1	0.6806 (2)	0.7176 (2)	0.54188 (5)	0.0226 (3)	
O3	0.6513 (2)	0.4530 (2)	0.66760 (5)	0.0351 (4)	
O4	0.1517 (2)	0.5956 (2)	0.59524 (5)	0.0219 (3)	
N3	0.4090 (3)	0.6598 (2)	0.68645 (5)	0.0204 (4)	
C2	0.6722 (3)	0.6736 (3)	0.58634 (7)	0.0206 (4)	
H2	0.7952	0.6604	0.6045	0.025*	
C3	0.5026 (3)	0.6467 (3)	0.60699 (6)	0.0181 (4)	
C4	0.3091 (3)	0.6483 (3)	0.58017 (6)	0.0176 (4)	
C4A	0.3200 (3)	0.7089 (3)	0.53230 (6)	0.0175 (4)	
C5	0.1473 (3)	0.7317 (3)	0.50257 (7)	0.0225 (4)	
H5	0.0196	0.7139	0.5136	0.027*	
C6	0.1615 (3)	0.7800 (3)	0.45738 (7)	0.0262 (5)	
H6	0.0437	0.7970	0.4376	0.031*	
C7	0.3497 (3)	0.8040 (3)	0.44074 (7)	0.0250 (5)	
H7	0.3586	0.8342	0.4095	0.030*	
C8	0.5213 (3)	0.7842 (3)	0.46923 (7)	0.0228 (4)	
H8	0.6488	0.8018	0.4581	0.027*	
C8A	0.5041 (3)	0.7376 (3)	0.51492 (6)	0.0184 (4)	
C31	0.5272 (3)	0.5798 (3)	0.65592 (7)	0.0218 (4)	
C32	0.4238 (4)	0.5723 (3)	0.73235 (7)	0.0329 (6)	
H32A	0.3328	0.6409	0.7513	0.049*	
H32B	0.3872	0.4331	0.7302	0.049*	
H32C	0.5612	0.5848	0.7461	0.049*	
C311	0.2721 (3)	0.8175 (3)	0.67737 (6)	0.0180 (4)	
C312	0.0764 (3)	0.7959 (4)	0.68895 (7)	0.0296 (5)	
H312	0.0338	0.6752	0.7011	0.036*	
C313	−0.0547 (4)	0.9506 (5)	0.68268 (8)	0.0428 (7)	
H313	−0.1879	0.9361	0.6908	0.051*	
C314	0.0046 (4)	1.1271 (4)	0.66470 (8)	0.0450 (8)	
H314	−0.0873	1.2330	0.6605	0.054*	
C315	0.1985 (4)	1.1481 (3)	0.65283 (8)	0.0352 (6)	
H315	0.2394	1.2679	0.6399	0.042*	
C316	0.3337 (3)	0.9941 (3)	0.65974 (6)	0.0225 (4)	
H316	0.4679	1.0098	0.6524	0.027*	

### (1) N-Methyl-4-oxo-N-phenyl-4H-chromene-3-carboxamide Atomic displacement parameters (Å^2^)

**Table:**

	*U*^11^	*U*^22^	*U*^33^	*U*^12^	*U*^13^	*U*^23^
O1	0.0171 (7)	0.0268 (8)	0.0242 (7)	0.0015 (6)	0.0046 (6)	−0.0019 (6)
O3	0.0367 (10)	0.0375 (9)	0.0303 (8)	0.0219 (8)	−0.0014 (7)	0.0022 (7)
O4	0.0176 (7)	0.0269 (8)	0.0215 (7)	−0.0015 (6)	0.0035 (5)	0.0009 (6)
N3	0.0232 (9)	0.0203 (8)	0.0178 (8)	0.0059 (7)	0.0024 (7)	0.0032 (6)
C2	0.0162 (10)	0.0218 (10)	0.0236 (10)	0.0045 (8)	−0.0001 (8)	−0.0030 (8)
C3	0.0151 (10)	0.0187 (9)	0.0206 (9)	0.0021 (8)	0.0018 (7)	−0.0025 (7)
C4	0.0164 (10)	0.0161 (9)	0.0203 (9)	0.0013 (8)	0.0024 (7)	−0.0019 (7)
C4A	0.0180 (10)	0.0153 (9)	0.0195 (9)	0.0008 (8)	0.0032 (7)	−0.0026 (7)
C5	0.0181 (10)	0.0257 (11)	0.0238 (10)	−0.0004 (9)	0.0025 (8)	−0.0009 (8)
C6	0.0254 (11)	0.0299 (12)	0.0227 (10)	0.0018 (10)	−0.0019 (8)	0.0010 (8)
C7	0.0343 (12)	0.0213 (10)	0.0202 (9)	−0.0002 (9)	0.0063 (9)	0.0007 (8)
C8	0.0254 (11)	0.0181 (10)	0.0263 (10)	0.0004 (9)	0.0101 (8)	−0.0027 (8)
C8A	0.0178 (10)	0.0148 (9)	0.0227 (9)	0.0012 (8)	0.0024 (8)	−0.0029 (7)
C31	0.0190 (10)	0.0226 (10)	0.0235 (10)	0.0049 (9)	−0.0005 (8)	0.0002 (8)
C32	0.0523 (16)	0.0281 (12)	0.0183 (10)	0.0063 (11)	0.0021 (10)	0.0045 (8)
C311	0.0160 (10)	0.0215 (10)	0.0162 (8)	0.0055 (8)	−0.0006 (7)	−0.0028 (7)
C312	0.0219 (11)	0.0415 (13)	0.0257 (10)	0.0011 (10)	0.0035 (9)	−0.0075 (9)
C313	0.0223 (12)	0.071 (2)	0.0347 (13)	0.0161 (13)	−0.0016 (10)	−0.0213 (13)
C314	0.0448 (16)	0.0515 (17)	0.0355 (13)	0.0353 (14)	−0.0166 (12)	−0.0195 (12)
C315	0.0542 (16)	0.0229 (11)	0.0257 (11)	0.0127 (11)	−0.0121 (11)	−0.0039 (9)
C316	0.0254 (11)	0.0232 (10)	0.0181 (9)	0.0021 (9)	−0.0023 (8)	−0.0017 (8)

### (1) N-Methyl-4-oxo-N-phenyl-4H-chromene-3-carboxamide Geometric parameters (Å, º)

**Table:**

O1—C2	1.348 (2)	C7—C8	1.374 (3)
O1—C8A	1.377 (2)	C7—H7	0.9500
O3—C31	1.229 (2)	C8—C8A	1.395 (3)
O4—C4	1.233 (2)	C8—H8	0.9500
N3—C31	1.361 (3)	C32—H32A	0.9800
N3—C311	1.425 (3)	C32—H32B	0.9800
N3—C32	1.472 (3)	C32—H32C	0.9800
C2—C3	1.347 (3)	C311—C316	1.386 (3)
C2—H2	0.9500	C311—C312	1.393 (3)
C3—C4	1.464 (3)	C312—C313	1.375 (3)
C3—C31	1.506 (3)	C312—H312	0.9500
C4—C4A	1.476 (3)	C313—C314	1.385 (4)
C4A—C8A	1.391 (3)	C313—H313	0.9500
C4A—C5	1.402 (3)	C314—C315	1.384 (4)
C5—C6	1.381 (3)	C314—H314	0.9500
C5—H5	0.9500	C315—C316	1.391 (3)
C6—C7	1.403 (3)	C315—H315	0.9500
C6—H6	0.9500	C316—H316	0.9500
			
C2—O1—C8A	118.54 (16)	O1—C8A—C8	116.18 (18)
C31—N3—C311	125.50 (16)	C4A—C8A—C8	122.26 (19)
C31—N3—C32	116.47 (17)	O3—C31—N3	121.05 (18)
C311—N3—C32	118.03 (17)	O3—C31—C3	119.82 (18)
C3—C2—O1	124.97 (19)	N3—C31—C3	119.13 (17)
C3—C2—H2	117.5	N3—C32—H32A	109.5
O1—C2—H2	117.5	N3—C32—H32B	109.5
C2—C3—C4	120.15 (18)	H32A—C32—H32B	109.5
C2—C3—C31	116.29 (18)	N3—C32—H32C	109.5
C4—C3—C31	122.62 (17)	H32A—C32—H32C	109.5
O4—C4—C3	123.61 (18)	H32B—C32—H32C	109.5
O4—C4—C4A	122.49 (18)	C316—C311—C312	120.02 (19)
C3—C4—C4A	113.82 (17)	C316—C311—N3	121.05 (18)
C8A—C4A—C5	118.01 (18)	C312—C311—N3	118.83 (19)
C8A—C4A—C4	120.47 (17)	C313—C312—C311	119.6 (2)
C5—C4A—C4	121.47 (18)	C313—C312—H312	120.2
C6—C5—C4A	120.51 (19)	C311—C312—H312	120.2
C6—C5—H5	119.7	C312—C313—C314	121.0 (2)
C4A—C5—H5	119.7	C312—C313—H313	119.5
C5—C6—C7	120.01 (19)	C314—C313—H313	119.5
C5—C6—H6	120.0	C315—C314—C313	119.5 (2)
C7—C6—H6	120.0	C315—C314—H314	120.2
C8—C7—C6	120.68 (19)	C313—C314—H314	120.2
C8—C7—H7	119.7	C314—C315—C316	120.1 (2)
C6—C7—H7	119.7	C314—C315—H315	119.9
C7—C8—C8A	118.51 (19)	C316—C315—H315	119.9
C7—C8—H8	120.7	C311—C316—C315	119.8 (2)
C8A—C8—H8	120.7	C311—C316—H316	120.1
O1—C8A—C4A	121.54 (17)	C315—C316—H316	120.1
			
C8A—O1—C2—C3	0.1 (3)	C7—C8—C8A—O1	179.39 (17)
O1—C2—C3—C4	5.2 (3)	C7—C8—C8A—C4A	0.7 (3)
O1—C2—C3—C31	174.45 (18)	C311—N3—C31—O3	174.8 (2)
C2—C3—C4—O4	168.55 (19)	C32—N3—C31—O3	−6.1 (3)
C31—C3—C4—O4	0.0 (3)	C311—N3—C31—C3	−6.0 (3)
C2—C3—C4—C4A	−8.3 (3)	C32—N3—C31—C3	173.13 (19)
C31—C3—C4—C4A	−176.85 (17)	C2—C3—C31—O3	−42.0 (3)
O4—C4—C4A—C8A	−169.94 (18)	C4—C3—C31—O3	126.9 (2)
C3—C4—C4A—C8A	7.0 (3)	C2—C3—C31—N3	138.8 (2)
O4—C4—C4A—C5	7.5 (3)	C4—C3—C31—N3	−52.3 (3)
C3—C4—C4A—C5	−175.54 (18)	C31—N3—C311—C316	−54.2 (3)
C8A—C4A—C5—C6	0.5 (3)	C32—N3—C311—C316	126.7 (2)
C4—C4A—C5—C6	−177.09 (19)	C31—N3—C311—C312	129.5 (2)
C4A—C5—C6—C7	0.9 (3)	C32—N3—C311—C312	−49.6 (3)
C5—C6—C7—C8	−1.4 (3)	C316—C311—C312—C313	0.1 (3)
C6—C7—C8—C8A	0.7 (3)	N3—C311—C312—C313	176.53 (19)
C2—O1—C8A—C4A	−1.6 (3)	C311—C312—C313—C314	0.5 (3)
C2—O1—C8A—C8	179.71 (17)	C312—C313—C314—C315	0.1 (4)
C5—C4A—C8A—O1	−179.89 (17)	C313—C314—C315—C316	−1.2 (3)
C4—C4A—C8A—O1	−2.3 (3)	C312—C311—C316—C315	−1.3 (3)
C5—C4A—C8A—C8	−1.2 (3)	N3—C311—C316—C315	−177.58 (18)
C4—C4A—C8A—C8	176.34 (18)	C314—C315—C316—C311	1.8 (3)

### (1) N-Methyl-4-oxo-N-phenyl-4H-chromene-3-carboxamide Hydrogen-bond geometry (Å, º)

**Table:**

*D*—H···*A*	*D*—H	H···*A*	*D*···*A*	*D*—H···*A*
C2—H2···O4^i^	0.95	2.47	3.253 (3)	140
C5—H5···O1^ii^	0.95	2.49	3.432 (3)	172
C314—H314···O3^iii^	0.95	2.33	3.255 (3)	164

Symmetry codes: (i) *x*+1, *y*, *z*; (ii) *x*−1, *y*, *z*; (iii) *x*−1, *y*+1, *z*.

### (3) 3-(Pyrrolidine-1-carbonyl)-4H-chromen-4-one Crystal data

**Table:**

C_14_H_13_NO_3_	*D*_x_ = 1.437 Mg m^−^^3^
*M**_r_* = 243.25	Mo *K*α radiation, λ = 0.71075 Å
Orthorhombic, *P*2_1_2_1_2_1_	Cell parameters from 4253 reflections
*a* = 7.430 (3) Å	θ = 2.3–31.2°
*b* = 11.963 (6) Å	µ = 0.10 mm^−^^1^
*c* = 12.648 (6) Å	*T* = 100 K
*V* = 1124.2 (9) Å^3^	Lath, colourless
*Z* = 4	0.22 × 0.07 × 0.03 mm
*F*(000) = 512	

### (3) 3-(Pyrrolidine-1-carbonyl)-4H-chromen-4-one Data collection

**Table:**

Rigaku Saturn724+ diffractometer	3294 independent reflections
Radiation source: Rotating Anode	3066 reflections with *I* > 2σ(*I*)
Confocal monochromator	*R*_int_ = 0.031
Detector resolution: 28.5714 pixels mm^-1^	θ_max_ = 31.3°, θ_min_ = 2.3°
profile data from ω–scans	*h* = −8→10
Absorption correction: multi-scan (*CrystalClear-SM Expert*; Rigaku, 2012)	*k* = −17→15
*T*_min_ = 0.978, *T*_max_ = 0.997	*l* = −18→18
7547 measured reflections	

### (3) 3-(Pyrrolidine-1-carbonyl)-4H-chromen-4-one Refinement

**Table:**

Refinement on *F*^2^	Hydrogen site location: inferred from neighbouring sites
Least-squares matrix: full	H-atom parameters constrained
*R*[*F*^2^ > 2σ(*F*^2^)] = 0.069	*w* = 1/[σ^2^(*F*_o_^2^) + (0.0459*P*)^2^ + 1.1472*P*] where *P* = (*F*_o_^2^ + 2*F*_c_^2^)/3
*wR*(*F*^2^) = 0.152	(Δ/σ)_max_ < 0.001
*S* = 1.07	Δρ_max_ = 0.51 e Å^−^^3^
3294 reflections	Δρ_min_ = −0.44 e Å^−^^3^
163 parameters	Absolute structure: Flack *x* determined using 946 quotients [(*I*^+^)-(*I*^-^)]/[(*I*^+^)+(*I*^-^)] (Parsons *et al*., 2013)
0 restraints	Absolute structure parameter: −0.1 (6)

### (3) 3-(Pyrrolidine-1-carbonyl)-4H-chromen-4-one Special details

**Table:**

Geometry. All e.s.d.'s (except the e.s.d. in the dihedral angle between two l.s. planes) are estimated using the full covariance matrix. The cell e.s.d.'s are taken into account individually in the estimation of e.s.d.'s in distances, angles and torsion angles; correlations between e.s.d.'s in cell parameters are only used when they are defined by crystal symmetry. An approximate (isotropic) treatment of cell e.s.d.'s is used for estimating e.s.d.'s involving l.s. planes.

### (3) 3-(Pyrrolidine-1-carbonyl)-4H-chromen-4-one Fractional atomic coordinates and isotropic or equivalent isotropic displacement parameters (Å^2^)

**Table:**

	*x*	*y*	*z*	*U*_iso_*/*U*_eq_	
O1	0.8069 (3)	0.5658 (2)	0.58566 (17)	0.0247 (5)	
O3	0.6707 (4)	0.8949 (2)	0.5280 (2)	0.0362 (6)	
O4	0.5779 (3)	0.66383 (19)	0.30612 (18)	0.0255 (5)	
N311	0.7972 (4)	0.8846 (2)	0.3665 (2)	0.0292 (6)	
C2	0.7960 (5)	0.6739 (3)	0.5573 (2)	0.0254 (7)	
H2	0.8374	0.7277	0.6068	0.030*	
C3	0.7316 (4)	0.7126 (3)	0.4646 (2)	0.0224 (6)	
C4	0.6548 (4)	0.6348 (3)	0.3885 (2)	0.0215 (6)	
C4A	0.6710 (4)	0.5165 (3)	0.4186 (2)	0.0212 (6)	
C5	0.6124 (4)	0.4305 (3)	0.3517 (2)	0.0236 (6)	
H5	0.5596	0.4485	0.2855	0.028*	
C6	0.6304 (5)	0.3197 (3)	0.3812 (3)	0.0263 (7)	
H6	0.5910	0.2620	0.3351	0.032*	
C7	0.7069 (5)	0.2928 (3)	0.4790 (3)	0.0271 (7)	
H7	0.7185	0.2166	0.4992	0.033*	
C8	0.7659 (5)	0.3762 (3)	0.5466 (3)	0.0259 (6)	
H8	0.8178	0.3581	0.6131	0.031*	
C8A	0.7474 (4)	0.4865 (3)	0.5153 (2)	0.0223 (6)	
C31	0.7301 (5)	0.8378 (3)	0.4541 (3)	0.0259 (6)	
C312	0.8921 (6)	0.8294 (3)	0.2780 (3)	0.0318 (8)	
H31A	0.8245	0.7634	0.2523	0.038*	
H31B	1.0147	0.8057	0.2991	0.038*	
C313	0.8989 (7)	0.9193 (3)	0.1957 (4)	0.0430 (10)	
H31C	0.7881	0.9192	0.1522	0.052*	
H31D	1.0043	0.9093	0.1487	0.052*	
C314	0.9147 (8)	1.0274 (4)	0.2587 (3)	0.0477 (12)	
H31E	0.8685	1.0916	0.2175	0.057*	
H31F	1.0414	1.0421	0.2784	0.057*	
C315	0.7994 (5)	1.0074 (3)	0.3571 (3)	0.0360 (8)	
H31G	0.8542	1.0424	0.4203	0.043*	
H31H	0.6763	1.0374	0.3474	0.043*	

### (3) 3-(Pyrrolidine-1-carbonyl)-4H-chromen-4-one Atomic displacement parameters (Å^2^)

**Table:**

	*U*^11^	*U*^22^	*U*^33^	*U*^12^	*U*^13^	*U*^23^
O1	0.0315 (12)	0.0273 (11)	0.0153 (9)	−0.0027 (10)	−0.0022 (9)	−0.0007 (8)
O3	0.0401 (14)	0.0323 (13)	0.0362 (14)	0.0008 (11)	0.0038 (12)	−0.0126 (11)
O4	0.0296 (12)	0.0265 (11)	0.0202 (10)	0.0029 (10)	−0.0052 (9)	0.0009 (9)
N311	0.0379 (16)	0.0207 (12)	0.0288 (14)	−0.0021 (12)	−0.0007 (13)	−0.0022 (11)
C2	0.0289 (16)	0.0275 (15)	0.0196 (14)	−0.0026 (13)	−0.0011 (13)	−0.0041 (12)
C3	0.0221 (14)	0.0245 (14)	0.0208 (14)	0.0004 (12)	0.0003 (12)	−0.0033 (11)
C4	0.0209 (13)	0.0247 (14)	0.0188 (13)	0.0017 (11)	0.0018 (11)	−0.0011 (11)
C4A	0.0195 (13)	0.0259 (14)	0.0180 (13)	−0.0005 (12)	0.0013 (11)	0.0002 (11)
C5	0.0246 (14)	0.0289 (15)	0.0174 (13)	−0.0008 (12)	0.0003 (12)	−0.0013 (11)
C6	0.0300 (17)	0.0246 (15)	0.0243 (15)	−0.0042 (12)	0.0012 (13)	−0.0017 (12)
C7	0.0325 (17)	0.0236 (14)	0.0252 (15)	−0.0005 (13)	0.0028 (14)	0.0053 (12)
C8	0.0274 (15)	0.0310 (16)	0.0193 (14)	0.0007 (13)	−0.0019 (13)	0.0044 (12)
C8A	0.0205 (14)	0.0292 (15)	0.0171 (13)	−0.0013 (12)	0.0008 (11)	−0.0012 (11)
C31	0.0267 (15)	0.0261 (15)	0.0250 (14)	−0.0003 (13)	−0.0040 (13)	−0.0043 (12)
C312	0.045 (2)	0.0271 (16)	0.0229 (15)	−0.0047 (16)	0.0005 (15)	0.0001 (13)
C313	0.047 (2)	0.042 (2)	0.040 (2)	0.0007 (19)	0.010 (2)	0.0069 (17)
C314	0.076 (3)	0.036 (2)	0.030 (2)	−0.019 (2)	−0.009 (2)	0.0046 (16)
C315	0.042 (2)	0.0226 (15)	0.044 (2)	−0.0042 (15)	−0.0086 (17)	0.0010 (14)

### (3) 3-(Pyrrolidine-1-carbonyl)-4H-chromen-4-one Geometric parameters (Å, º)

**Table:**

O1—C2	1.345 (4)	C6—H6	0.9500
O1—C8A	1.374 (4)	C7—C8	1.386 (5)
O3—C31	1.239 (4)	C7—H7	0.9500
O4—C4	1.238 (4)	C8—C8A	1.384 (4)
N311—C31	1.337 (4)	C8—H8	0.9500
N311—C315	1.474 (4)	C312—C313	1.498 (5)
N311—C312	1.480 (5)	C312—H31A	0.9900
C2—C3	1.348 (4)	C312—H31B	0.9900
C2—H2	0.9500	C313—C314	1.524 (6)
C3—C4	1.455 (4)	C313—H31C	0.9900
C3—C31	1.504 (4)	C313—H31D	0.9900
C4—C4A	1.471 (4)	C314—C315	1.529 (6)
C4A—C8A	1.396 (4)	C314—H31E	0.9900
C4A—C5	1.401 (4)	C314—H31F	0.9900
C5—C6	1.383 (5)	C315—H31G	0.9900
C5—H5	0.9500	C315—H31H	0.9900
C6—C7	1.399 (5)		
			
C2—O1—C8A	118.2 (2)	O1—C8A—C4A	121.4 (3)
C31—N311—C315	119.2 (3)	C8—C8A—C4A	122.4 (3)
C31—N311—C312	128.2 (3)	O3—C31—N311	121.8 (3)
C315—N311—C312	112.2 (3)	O3—C31—C3	119.0 (3)
O1—C2—C3	125.7 (3)	N311—C31—C3	119.2 (3)
O1—C2—H2	117.2	N311—C312—C313	102.8 (3)
C3—C2—H2	117.2	N311—C312—H31A	111.2
C2—C3—C4	119.7 (3)	C313—C312—H31A	111.2
C2—C3—C31	114.9 (3)	N311—C312—H31B	111.2
C4—C3—C31	125.1 (3)	C313—C312—H31B	111.2
O4—C4—C3	124.0 (3)	H31A—C312—H31B	109.1
O4—C4—C4A	121.7 (3)	C312—C313—C314	104.4 (3)
C3—C4—C4A	114.3 (3)	C312—C313—H31C	110.9
C8A—C4A—C5	117.8 (3)	C314—C313—H31C	110.9
C8A—C4A—C4	120.5 (3)	C312—C313—H31D	110.9
C5—C4A—C4	121.7 (3)	C314—C313—H31D	110.9
C6—C5—C4A	120.7 (3)	H31C—C313—H31D	108.9
C6—C5—H5	119.7	C313—C314—C315	104.5 (3)
C4A—C5—H5	119.7	C313—C314—H31E	110.9
C5—C6—C7	119.9 (3)	C315—C314—H31E	110.9
C5—C6—H6	120.0	C313—C314—H31F	110.9
C7—C6—H6	120.0	C315—C314—H31F	110.9
C8—C7—C6	120.6 (3)	H31E—C314—H31F	108.9
C8—C7—H7	119.7	N311—C315—C314	103.2 (3)
C6—C7—H7	119.7	N311—C315—H31G	111.1
C8A—C8—C7	118.6 (3)	C314—C315—H31G	111.1
C8A—C8—H8	120.7	N311—C315—H31H	111.1
C7—C8—H8	120.7	C314—C315—H31H	111.1
O1—C8A—C8	116.2 (3)	H31G—C315—H31H	109.1
			
C8A—O1—C2—C3	−0.2 (5)	C5—C4A—C8A—O1	−179.5 (3)
O1—C2—C3—C4	4.3 (5)	C4—C4A—C8A—O1	1.0 (4)
O1—C2—C3—C31	178.5 (3)	C5—C4A—C8A—C8	−0.3 (5)
C2—C3—C4—O4	173.1 (3)	C4—C4A—C8A—C8	−179.8 (3)
C31—C3—C4—O4	−0.4 (5)	C315—N311—C31—O3	−0.5 (5)
C2—C3—C4—C4A	−5.3 (4)	C312—N311—C31—O3	171.9 (3)
C31—C3—C4—C4A	−178.8 (3)	C315—N311—C31—C3	−178.7 (3)
O4—C4—C4A—C8A	−175.6 (3)	C312—N311—C31—C3	−6.3 (5)
C3—C4—C4A—C8A	2.8 (4)	C2—C3—C31—O3	−46.6 (4)
O4—C4—C4A—C5	4.9 (5)	C4—C3—C31—O3	127.2 (4)
C3—C4—C4A—C5	−176.7 (3)	C2—C3—C31—N311	131.6 (3)
C8A—C4A—C5—C6	−0.1 (5)	C4—C3—C31—N311	−54.6 (5)
C4—C4A—C5—C6	179.4 (3)	C31—N311—C312—C313	168.7 (4)
C4A—C5—C6—C7	0.4 (5)	C315—N311—C312—C313	−18.4 (4)
C5—C6—C7—C8	−0.4 (5)	N311—C312—C313—C314	32.8 (5)
C6—C7—C8—C8A	0.0 (5)	C312—C313—C314—C315	−36.0 (5)
C2—O1—C8A—C8	178.2 (3)	C31—N311—C315—C314	169.9 (3)
C2—O1—C8A—C4A	−2.5 (4)	C312—N311—C315—C314	−3.6 (4)
C7—C8—C8A—O1	179.6 (3)	C313—C314—C315—N311	24.0 (4)
C7—C8—C8A—C4A	0.3 (5)		

### (3) 3-(Pyrrolidine-1-carbonyl)-4H-chromen-4-one Hydrogen-bond geometry (Å, º)

Cg is the centroid of the benzene ring C4*A*/C5–C8/C8*A*

**Table:**

*D*—H···*A*	*D*—H	H···*A*	*D*···*A*	*D*—H···*A*
C312—H31*A*···O4	0.99	2.29	3.082 (5)	136
C2—H2···O4^i^	0.95	2.47	3.338 (4)	152
C6—H6···O4^ii^	0.95	2.48	3.389 (4)	161
C8—H8···O4^iii^	0.95	2.57	3.514 (4)	170
C314—H31*E*···O3^iv^	0.99	2.42	3.128 (5)	128
C313—H31*D*···*Cg*^v^	0.99	2.59	3.570 (6)	170

Symmetry codes: (i) *x*+1/2, −*y*+3/2, −*z*+1; (ii) −*x*+1, *y*−1/2, −*z*+1/2; (iii) −*x*+3/2, −*y*+1, *z*+1/2; (iv) −*x*+3/2, −*y*+2, *z*−1/2; (v) *x*−1/2, −*y*+3/2, −*z*+1.

### (4) 2-(Pyrrolidine-1-carbonyl)-4H-chromen-4-one Crystal data

**Table:**

C_14_H_13_NO_3_	*D*_x_ = 1.411 Mg m^−^^3^
*M**_r_* = 243.25	Mo *K*α radiation, λ = 0.71075 Å
Orthorhombic, *A**b**a*2	Cell parameters from 3760 reflections
*a* = 15.337 (6) Å	θ = 2.3–31.2°
*b* = 21.940 (8) Å	µ = 0.10 mm^−^^1^
*c* = 6.808 (3) Å	*T* = 100 K
*V* = 2290.8 (16) Å^3^	Needle, colourless
*Z* = 8	0.50 × 0.04 × 0.02 mm
*F*(000) = 1024	

### (4) 2-(Pyrrolidine-1-carbonyl)-4H-chromen-4-one Data collection

**Table:**

Rigaku Saturn724+ diffractometer	3374 independent reflections
Radiation source: Rotating Anode	3079 reflections with *I* > 2σ(*I*)
Confocal monochromator	*R*_int_ = 0.034
Detector resolution: 28.5714 pixels mm^-1^	θ_max_ = 31.3°, θ_min_ = 2.3°
profile data from ω–scans	*h* = −21→17
Absorption correction: multi-scan (*CrystalClear-SM Expert*; Rigaku, 2012)	*k* = −29→31
*T*_min_ = 0.952, *T*_max_ = 0.998	*l* = −9→8
9598 measured reflections	

### (4) 2-(Pyrrolidine-1-carbonyl)-4H-chromen-4-one Refinement

**Table:**

Refinement on *F*^2^	Hydrogen site location: inferred from neighbouring sites
Least-squares matrix: full	H-atom parameters constrained
*R*[*F*^2^ > 2σ(*F*^2^)] = 0.048	*w* = 1/[σ^2^(*F*_o_^2^) + (0.0386*P*)^2^ + 1.0329*P*] where *P* = (*F*_o_^2^ + 2*F*_c_^2^)/3
*wR*(*F*^2^) = 0.102	(Δ/σ)_max_ < 0.001
*S* = 1.11	Δρ_max_ = 0.32 e Å^−^^3^
3374 reflections	Δρ_min_ = −0.21 e Å^−^^3^
163 parameters	Absolute structure: Flack *x* determined using 1043 quotients [(*I*^+^)-(*I*^-^)]/[(*I*^+^)+(*I*^-^)] (Parsons *et al*., 2013)
1 restraint	Absolute structure parameter: 1.2 (5)

### (4) 2-(Pyrrolidine-1-carbonyl)-4H-chromen-4-one Special details

**Table:**

Geometry. All e.s.d.'s (except the e.s.d. in the dihedral angle between two l.s. planes) are estimated using the full covariance matrix. The cell e.s.d.'s are taken into account individually in the estimation of e.s.d.'s in distances, angles and torsion angles; correlations between e.s.d.'s in cell parameters are only used when they are defined by crystal symmetry. An approximate (isotropic) treatment of cell e.s.d.'s is used for estimating e.s.d.'s involving l.s. planes.

### (4) 2-(Pyrrolidine-1-carbonyl)-4H-chromen-4-one Fractional atomic coordinates and isotropic or equivalent isotropic displacement parameters (Å^2^)

**Table:**

	*x*	*y*	*z*	*U*_iso_*/*U*_eq_	
O1	0.60596 (8)	0.22901 (6)	0.4803 (2)	0.0186 (3)	
O2	0.42149 (9)	0.32316 (7)	0.4657 (3)	0.0254 (4)	
O4	0.42290 (10)	0.09521 (7)	0.4038 (3)	0.0244 (4)	
C2	0.52132 (12)	0.24216 (9)	0.4484 (3)	0.0176 (4)	
C3	0.45851 (13)	0.19980 (9)	0.4233 (3)	0.0193 (4)	
H3	0.3999	0.2124	0.4038	0.023*	
C4	0.47910 (13)	0.13519 (9)	0.4255 (3)	0.0191 (4)	
C4A	0.57164 (13)	0.12141 (9)	0.4559 (3)	0.0182 (4)	
C5	0.60362 (13)	0.06129 (9)	0.4609 (4)	0.0207 (4)	
H5	0.5647	0.0281	0.4423	0.025*	
C6	0.69058 (14)	0.05020 (9)	0.4923 (4)	0.0227 (5)	
H6	0.7113	0.0094	0.4973	0.027*	
C7	0.74912 (15)	0.09874 (9)	0.5172 (4)	0.0220 (4)	
H7	0.8093	0.0906	0.5375	0.026*	
C8	0.71978 (14)	0.15805 (9)	0.5122 (3)	0.0203 (4)	
H8	0.7592	0.1911	0.5286	0.024*	
C8A	0.63128 (13)	0.16867 (9)	0.4826 (3)	0.0174 (4)	
C21	0.49892 (13)	0.30951 (9)	0.4482 (3)	0.0186 (4)	
N211	0.56212 (11)	0.35131 (7)	0.4300 (3)	0.0185 (4)	
C212	0.53740 (13)	0.41656 (8)	0.4348 (4)	0.0203 (4)	
H21G	0.5017	0.4259	0.5522	0.024*	
H21H	0.5043	0.4280	0.3156	0.024*	
C213	0.62437 (14)	0.44949 (9)	0.4428 (4)	0.0233 (5)	
H21E	0.6442	0.4548	0.5801	0.028*	
H21F	0.6207	0.4899	0.3789	0.028*	
C214	0.68522 (14)	0.40700 (10)	0.3296 (4)	0.0231 (5)	
H21C	0.6780	0.4123	0.1861	0.028*	
H21D	0.7469	0.4146	0.3648	0.028*	
C215	0.65665 (13)	0.34367 (9)	0.3941 (4)	0.0208 (4)	
H21A	0.6676	0.3132	0.2896	0.025*	
H21B	0.6874	0.3309	0.5152	0.025*	

### (4) 2-(Pyrrolidine-1-carbonyl)-4H-chromen-4-one Atomic displacement parameters (Å^2^)

**Table:**

	*U*^11^	*U*^22^	*U*^33^	*U*^12^	*U*^13^	*U*^23^
O1	0.0133 (6)	0.0152 (6)	0.0271 (8)	−0.0003 (5)	−0.0021 (6)	0.0001 (7)
O2	0.0154 (7)	0.0248 (7)	0.0360 (10)	0.0030 (5)	−0.0001 (7)	0.0003 (8)
O4	0.0199 (8)	0.0242 (7)	0.0289 (10)	−0.0073 (6)	0.0007 (7)	−0.0016 (7)
C2	0.0166 (9)	0.0184 (8)	0.0177 (10)	0.0008 (7)	0.0000 (8)	−0.0008 (9)
C3	0.0155 (9)	0.0211 (9)	0.0213 (11)	−0.0005 (7)	0.0013 (8)	0.0010 (10)
C4	0.0191 (10)	0.0209 (9)	0.0173 (11)	−0.0035 (7)	0.0021 (8)	−0.0006 (9)
C4A	0.0188 (9)	0.0181 (8)	0.0177 (11)	−0.0024 (7)	0.0028 (8)	0.0002 (9)
C5	0.0212 (10)	0.0175 (8)	0.0235 (11)	−0.0027 (7)	0.0028 (9)	0.0009 (9)
C6	0.0245 (11)	0.0167 (9)	0.0267 (12)	0.0018 (7)	0.0039 (9)	0.0013 (10)
C7	0.0185 (10)	0.0230 (10)	0.0244 (12)	0.0018 (8)	0.0010 (9)	0.0030 (10)
C8	0.0179 (10)	0.0197 (9)	0.0233 (11)	−0.0014 (7)	−0.0008 (8)	0.0002 (9)
C8A	0.0172 (9)	0.0161 (8)	0.0189 (10)	0.0002 (7)	−0.0004 (8)	0.0009 (8)
C21	0.0174 (9)	0.0199 (9)	0.0185 (11)	0.0022 (7)	−0.0011 (8)	−0.0006 (9)
N211	0.0160 (8)	0.0163 (7)	0.0231 (10)	0.0013 (6)	−0.0010 (7)	−0.0011 (8)
C212	0.0216 (10)	0.0167 (9)	0.0225 (11)	0.0034 (7)	−0.0021 (9)	0.0005 (9)
C213	0.0242 (10)	0.0165 (9)	0.0291 (13)	−0.0021 (7)	−0.0011 (9)	0.0011 (10)
C214	0.0190 (11)	0.0238 (11)	0.0263 (12)	−0.0028 (8)	−0.0009 (9)	0.0022 (10)
C215	0.0143 (9)	0.0185 (9)	0.0295 (12)	0.0004 (7)	−0.0004 (8)	0.0008 (9)

### (4) 2-(Pyrrolidine-1-carbonyl)-4H-chromen-4-one Geometric parameters (Å, º)

**Table:**

O1—C2	1.347 (2)	C8—C8A	1.392 (3)
O1—C8A	1.380 (2)	C8—H8	0.9500
O2—C21	1.230 (2)	C21—N211	1.340 (3)
O4—C4	1.239 (2)	N211—C215	1.480 (3)
C2—C3	1.349 (3)	N211—C212	1.481 (2)
C2—C21	1.517 (3)	C212—C213	1.518 (3)
C3—C4	1.452 (3)	C212—H21G	0.9900
C3—H3	0.9500	C212—H21H	0.9900
C4—C4A	1.466 (3)	C213—C214	1.528 (3)
C4A—C8A	1.395 (3)	C213—H21E	0.9900
C4A—C5	1.408 (3)	C213—H21F	0.9900
C5—C6	1.373 (3)	C214—C215	1.522 (3)
C5—H5	0.9500	C214—H21C	0.9900
C6—C7	1.403 (3)	C214—H21D	0.9900
C6—H6	0.9500	C215—H21A	0.9900
C7—C8	1.377 (3)	C215—H21B	0.9900
C7—H7	0.9500		
			
C2—O1—C8A	118.59 (15)	O2—C21—C2	117.10 (17)
O1—C2—C3	124.10 (18)	N211—C21—C2	120.19 (17)
O1—C2—C21	115.27 (16)	C21—N211—C215	130.26 (16)
C3—C2—C21	120.60 (17)	C21—N211—C212	118.31 (16)
C2—C3—C4	121.05 (19)	C215—N211—C212	111.34 (15)
C2—C3—H3	119.5	N211—C212—C213	103.65 (16)
C4—C3—H3	119.5	N211—C212—H21G	111.0
O4—C4—C3	122.60 (19)	C213—C212—H21G	111.0
O4—C4—C4A	122.99 (19)	N211—C212—H21H	111.0
C3—C4—C4A	114.41 (17)	C213—C212—H21H	111.0
C8A—C4A—C5	117.71 (18)	H21G—C212—H21H	109.0
C8A—C4A—C4	120.01 (18)	C212—C213—C214	103.19 (17)
C5—C4A—C4	122.28 (17)	C212—C213—H21E	111.1
C6—C5—C4A	120.57 (18)	C214—C213—H21E	111.1
C6—C5—H5	119.7	C212—C213—H21F	111.1
C4A—C5—H5	119.7	C214—C213—H21F	111.1
C5—C6—C7	120.39 (19)	H21E—C213—H21F	109.1
C5—C6—H6	119.8	C215—C214—C213	103.63 (19)
C7—C6—H6	119.8	C215—C214—H21C	111.0
C8—C7—C6	120.3 (2)	C213—C214—H21C	111.0
C8—C7—H7	119.8	C215—C214—H21D	111.0
C6—C7—H7	119.8	C213—C214—H21D	111.0
C7—C8—C8A	118.71 (19)	H21C—C214—H21D	109.0
C7—C8—H8	120.6	N211—C215—C214	103.08 (17)
C8A—C8—H8	120.6	N211—C215—H21A	111.1
O1—C8A—C8	115.90 (17)	C214—C215—H21A	111.1
O1—C8A—C4A	121.82 (17)	N211—C215—H21B	111.1
C8—C8A—C4A	122.28 (18)	C214—C215—H21B	111.1
O2—C21—N211	122.71 (18)	H21A—C215—H21B	109.1
			
C8A—O1—C2—C3	1.8 (3)	C5—C4A—C8A—O1	−179.9 (2)
C8A—O1—C2—C21	179.99 (19)	C4—C4A—C8A—O1	−0.3 (3)
O1—C2—C3—C4	−1.2 (4)	C5—C4A—C8A—C8	0.4 (3)
C21—C2—C3—C4	−179.3 (2)	C4—C4A—C8A—C8	180.0 (2)
C2—C3—C4—O4	179.6 (2)	O1—C2—C21—O2	−163.5 (2)
C2—C3—C4—C4A	−0.2 (3)	C3—C2—C21—O2	14.8 (3)
O4—C4—C4A—C8A	−178.9 (2)	O1—C2—C21—N211	16.3 (3)
C3—C4—C4A—C8A	0.9 (3)	C3—C2—C21—N211	−165.4 (2)
O4—C4—C4A—C5	0.6 (4)	O2—C21—N211—C215	−175.3 (2)
C3—C4—C4A—C5	−179.5 (2)	C2—C21—N211—C215	5.0 (4)
C8A—C4A—C5—C6	0.4 (4)	O2—C21—N211—C212	1.1 (3)
C4—C4A—C5—C6	−179.1 (2)	C2—C21—N211—C212	−178.7 (2)
C4A—C5—C6—C7	−0.9 (4)	C21—N211—C212—C213	171.5 (2)
C5—C6—C7—C8	0.6 (4)	C215—N211—C212—C213	−11.5 (3)
C6—C7—C8—C8A	0.1 (3)	N211—C212—C213—C214	31.0 (2)
C2—O1—C8A—C8	178.7 (2)	C212—C213—C214—C215	−39.4 (2)
C2—O1—C8A—C4A	−1.0 (3)	C21—N211—C215—C214	163.8 (2)
C7—C8—C8A—O1	179.6 (2)	C212—N211—C215—C214	−12.8 (3)
C7—C8—C8A—C4A	−0.7 (3)	C213—C214—C215—N211	31.8 (2)

### (4) 2-(Pyrrolidine-1-carbonyl)-4H-chromen-4-one Hydrogen-bond geometry (Å, º)

**Table:**

*D*—H···*A*	*D*—H	H···*A*	*D*···*A*	*D*—H···*A*
C8—H8···O2^i^	0.95	2.55	3.137 (3)	121
C214—H21*C*···O4^ii^	0.99	2.47	3.340 (3)	146

Symmetry codes: (i) *x*+1/2, −*y*+1/2, *z*; (ii) −*x*+1, −*y*+1/2, *z*−1/2.
